# Urology practice during the COVID-19 vaccination campaign

**DOI:** 10.1177/03915603211016321

**Published:** 2021-05-13

**Authors:** Vincenzo Ficarra, Giacomo Novara, Gianluca Giannarini, Cosimo De Nunzio, Alberto Abrate, Riccardo Bartoletti, Alessandro Crestani, Francesco Esperto, Antonio Galfano, Andrea Gregori, Giovanni Liguori, Nicola Pavan, Alchiede Simonato, Carlo Trombetta, Andrea Tubaro, Francesco Porpiglia, Roberto Mario Scarpa, Vincenzo Mirone

**Affiliations:** 1 Department of Human and Pediatric Pathology “Gaetano Barresi”, Urology Section, University of Messina, Messina, Italy; 2 Department of Surgery, Oncology, and Gastroenterology, Urology Clinic, University of Padua, Padua, Italy; 3 Urology Unit, “Santa Maria della Misericordia” University Hospital, Udine, Italy; 4 Department of Urology, Sant’Andrea Hospital, Sapienza University of Rome, Roma, Italy; 5 Department of Surgical, Oncological and Oral Sciences, Urology Section, University of Palermo, Palermo, Italy; 6 Department of Translational Research and New Technologies, Urology Unit, University of Pisa, Pisa, Italy; 7 Urology Unit, Veneto Institute of Oncology IOV-IRCCS, Padua, Italy; 8 Department of Urology, Campus Bio-Medico University of Rome, Rome, Italy; 9 Urology Unit, ASST Grande Ospedale Metropolitano Niguarda, Milan, Italy; 10 Urology Unit, ASST Fatebenefratelli Sacco, Sacco Hospital, Milan, Italy; 11 Department of Urology, Cattinara Hospital, University of Trieste, Trieste, Italy; 12 Department of Oncology, Division of Urology, School of Medicine, University of Turin, San Luigi Hospital, Orbassano, Turin, Italy; 13 Department of Urology, Federico II University of Naples, Naples, Italy

**Keywords:** Coronavirus, COVID-19, pandemic, vaccine, urology, clinical practice guidelines, surgery, endourology

## Abstract

**Introduction:**

The current scenario of the COVID-19 pandemic is significantly different from that of the first, emergency phase. Several countries in the world are experiencing a second, or even a third, wave of contagion, while awaiting the effects of mass vaccination campaigns. The aim of this report was to provide an update of previously released recommendations on prioritization and restructuring of urological activities.

**Methods:**

A large group of Italian urologists directly involved in the reorganization of their urological wards during the first and second phase of the pandemic agreed on a set of updated recommendations for current urology practice.

**Results:**

The updated recommendations included strategies for the prioritization of both surgical and outpatient activities, implementation of perioperative pathways for patients scheduled for elective surgery, management of urological conditions in infected patients. Future scenarios with possible implementation of telehealth and reshaping of clinical practice following the effects of vaccination are also discussed.

**Conclusion:**

The present update may be a valid tool to be used in the clinical practice, may provide useful recommendations for national and international urological societies, and may be a cornerstone for further discussion on the topic, also considering further evolution of the pandemic after the recently initiated mass vaccination campaigns.

## Introduction

Starting from February 2020, the coronavirus disease 2019 (COVID-19) pandemic, caused by the severe acute respiratory syndrome coronavirus 2 (SARS-CoV-2), has generated a rapid and tragic health emergency worldwide due to the need to assist an overwhelming number of infected patients with severe acute respiratory syndrome requiring mechanical ventilation in intensive care units. Simultaneously to aggressive containment efforts implemented by the national political and health authorities, suspension of all outpatient and non-urgent activities coupled with restrictions in scheduling non-deferrable and urgent interventions has determined a major reorganization of all surgical activities, mainly depending on the availability of anesthesiologists, mechanical ventilators, and hospital beds.

In this war scenario, in March 2020 a large team of Italian experts affiliated to the Research Urology Network drafted a document with the purpose to provide a framework to reorganize urological activities, and to identify the procedures to prioritize in the management of the most common urological conditions during the emergency phase of the COVID-19 pandemic.^1^ This together with other expert-opinion documents that have classified urologic procedures in deferrable, semi-deferrable, non-deferrable, and urgent according to disease prognosis, have been widely cited in the peer-reviewed literature, and also considered in the drafting of several national and international guidelines published during the emergency phase of the pandemic.^1–4^

The end of the first lockdown, which took place in May 2020 in most European countries, marked the end of the emergency phase and the start of second phase of the pandemic, characterized by a decrease in contagion index across most countries. Urological activities gradually returned to an acceptable status in all geographic areas. Several political and economic interventions were planned to increase the human and technical resources needed to sustain a possible second wave of contagion, which indeed started from October 2020. For this reason, new restrictive measures were implemented in most European countries, which ultimately reduced the impact of the second wave on health systems of most countries, and, consequently, on the urologic activities in most COVID-19 hospitals. Obviously, the heterogeneous patterns of virus diffusion across different countries may be responsible for a variable impact on the urological activities.

Considering that the current scenario is significantly different from that of the first phase, and predicting a possible third wave while awaiting the implementation of mass vaccination campaigns,^5,6^ we believe that the urological community needs an update of previous recommendations on priority and reorganization of urologic activities. The present document is based on the opinion and experience maturated by the panel members in the management of urological conditions during the first and second phase of the pandemic. The panel scheduled to update their previous document at the end of December 2020. The final version of the present document was approved unanimously on January 10, 2021.

## Proposed management of urological conditions in SARS-CoV-2-negative patients

### Urgent procedures

Table 1 summarizes the procedures that should be performed in patients with urgent urological conditions. Considering the increasing availability of anesthesiologists and ventilators during the second phase of the pandemic, it is no longer needed to favor procedures that can be performed under local anesthesia instead of those that are routinely performed using regional or general anesthesia. Consequently, in the management of upper urinary tract obstruction, the choice between placing a ureteral stent (under regional or general anesthesia) or a percutaneous nephrostomy tube (under local anesthesia) to drain the upper urinary tract should be made according to the appropriate clinical circumstances and/or surgeon and patient preferences. Additionally, management of gross hematuria and genitourinary traumata should continue to follow the recommendations of the international guidelines.

**Table 1.** Urgent and emergent urological conditions with proposed treatment options during the COVID-19 vaccination campaign.

Condition	Treatment options
Upper urinary tract obstruction or infection	Nephrostomy tube
	Stent placement under anesthesia
Acute urinary retention	Urethral catheter or suprapubic tube
Clot retention	Clot evacuation and eventual concomitant hemostatic transurethral resection of bladder cancer or prostate
Urinary tract trauma	Treatment according to international guidelines:
	Monitoring and/or endovascular treatment
	Surgical treatment (hemodynamic instability or major polytrauma)
Spermatic cord torsion	Manual derotation
	Surgical exploration and orchidopexy
Infection of artificial urinary sphincter or penile prosthesis	Explant of the infected device
Scrotal abscesses, Fournier’s gangrene	Drainage
	Surgical treatment
Priapism	Corpora cavernosal aspiration/irrigation under local anesthesia
	Shunt

The panel suggests that all possible patient-related factors and comorbidities should be taken into account when triaging urgent conditions and planning corresponding treatments.

### Procedures for oncological conditions

According to the current situation and the experience matured out of the emergency phase of the pandemic, the panel strongly disagrees on the opportunity to replace some surgical treatments for urological malignancies with other treatments that do not require anesthesia. Alternative treatments to surgery should be taken into consideration in the decision-making process according to international guidelines and independently from the COVID-19 pandemic. Therefore, the opinion of the panel is that all efforts should be made during the second and third phase of the pandemic to deliver appropriate treatments in order not to jeopardize cancer-related outcomes and quality of life.

Similarly to the recommendations of the European Association of Urology Guidelines Office Rapid Reaction Group (EAU GORRG),^2^ urologic procedures for cancers have been classified in urgent (<24 h), non-deferrable (4–6 weeks), semi-deferrable (6–12 weeks) and deferrable (>12 weeks). All procedures for which a delay can be detrimental to oncological outcomes should be considered non-deferrable. Table 2 summarizes the panel recommendations concerning the priority to be assigned to the main surgical procedures to treat urological malignancies.

**Table 2.** Proposed priority of surgical procedures for the management of urological malignancies during the COVID-19 vaccination campaign.

Malignancy	Condition	Surgical procedure	Priority
Bladder	Intractable hematuria	Hemostatic TURBT	Urgent (<24 h)
		Palliative cystectomy	
	Muscle-invasive bladder cancer	Radical cystectomy and urinary diversion (continent/incontinent)	Non-deferrable (4–6 weeks)
	High-risk non-muscle-invasive bladder cancer		
	High-risk non-muscle invasive bladder cancer not suitable to, or refusing, intravesical BCG	Radical cystectomy and urinary diversion (continent/incontinent)	Semi-deferrable (6–12 weeks)
	High-grade cTx bladder cancer	TURBT	Non-deferrable (4–6 weeks)
	High-grade cT1/CIS candidate to repeat TURBT		
	Bladder tumor >2 cm on first diagnosis or recurrence	TURBT	Non-deferrable (4–6 weeks)
	Bladder tumor <2 cm on first diagnosis or recurrence (previous Ta low grade)	TURBT	Semi-deferrable (6–12 weeks)
Kidney (parenchymal)	Intractable tumor mass bleeding	Percutaneous embolization	Urgent (<24 h)
		Radical nephrectomy	
	cT3-4 tumor	Radical nephrectomy ± tumor thrombectomy	Non-deferrable (4–6 weeks)
	cT2 tumor or cystic mass Bosniak category 4	Radical nephrectomy	Non-deferrable (4–6 weeks)
		Partial nephrectomy in very selected cases	
	cT1b tumor or cystic mass Bosniak category 4	Partial or radical nephrectomy	Semi-deferrable (6–12 weeks)
	cT1a tumor or cystic mass Bosniak category 3 or 4	Partial nephrectomy	Deferrable (>12 weeks)
		Radical nephrectomy in very selected cases	
Upper urinary tract	Intractable hematuria	Palliative nephroureterectomy	Urgent (<24 h)
	High grade ⩾ cT1 urothelial cancer	Nephroureterectomy with eventual concomitant lymph node dissection	Non-deferrable (4–6 weeks)
Prostate	High risk localized or locally advancer prostate cancer not suitable to, or refusing, radiation therapy, or preferring surgery in the context of a multimodality treatment	Radical prostatectomy with pelvic lymph node dissection	Non-deferrable (4–6 weeks)
	Intermediate risk, localized prostate cancer	Radical prostatectomy with pelvic lymph node dissection	Semi-deferrable (6–12 weeks)
	Low risk, localized prostate cancer	Radical prostatectomy with pelvic lymph node dissection	Deferrable (>12 weeks)
Testis	Testicular mass highly suspicious for cancer	Radical orchidectomy	Non-deferrable (4–6 weeks)
	Post-chemotherapy residual retroperitoneal mass	Retroperitoneal lymph node dissection	Non-deferrable (4–6 weeks)
Penis	> cT1G3 penile cancer	Partial or total penectomy	Non-deferrable (4–6 weeks)
		Inguinal lymph node dissection (when indicated by international guidelines)	

BCG: Bacillus Calmette-Guerin; TURBT: transurethral resection of bladder tumor.

Concerning bladder cancer, we advocate to urgently (<24 h) proceed to transurethral resection or palliative cystectomy in the presence of intractable hematuria due to bladder cancer rather than to immediate radiotherapy ± chemotherapy as previously recommended by the EAU GORRG.^2^ Radical cystectomy should be still considered the gold standard treatment of muscle-invasive bladder cancer, ad regarded as a non-deferrable procedure. Moreover, we strongly support the option to treat patients, too, with high-risk non-muscle invasive bladder cancer that are unresponsive to intravesical Bacillus Calmette-Guerin (BCG), within 4–6 weeks.

As for kidney cancer, while surgical treatment for cT2-4 renal masses should be still considered as non-deferrable, management of cT1a and cT1b renal masses can be postponed to more than 12 weeks and 6–12 weeks, respectively. Although international guidelines support the use of active surveillance and ablative percutaneous treatment as an alternative to surgery in the treatment of selected patients with cT1a parenchymal renal tumors, we think that in this phase the final decision should not be influenced by the COVID-19 pandemic.

As for prostate cancer, the panel still considers radical prostatectomy with pelvic lymph-node dissection as non-deferrable in high-risk patients with localized disease or in those with locally advanced disease who are not suitable for, or refuse, radiation therapy ± androgen deprivation therapy. Radical prostatectomy ± pelvic lymph-node dissection can be considered as a semi-deferrable or deferrable procedure in patients with intermediate- or low-risk disease.

The Authors still suggest that when planning surgical procedures that are considered non-deferrable from the oncological standpoint, other considerations should be made, mainly with regard to the availability of intensive care for possible postoperative assistance. Centralization of more complex surgical procedures to high-volume centers is a strongly recommended measure to minimize the need for postoperative intensive care in the second and third phase of the pandemic.

### Procedures for benign conditions

In the previous document, all the procedures to treat benign diseases were considered deferrable until the end of the emergency phase.^1^ Currently, some months after the end of the emergency phase, many patients with benign urological conditions are still waiting for surgical treatment. Table 3 summarizes the recommendations on the priority of surgical and endoscopic procedures to treat the most common benign urological conditions during the second and third pandemic phase.

**Table 3.** Proposed priority for the management of urological benign diseases during the COVID-19 vaccination campaign.

Benign disease	Condition	Complications	Priority
Stones	Obstructing ureteral or renal stone	Infection	Urgent (<24 h)
		Solitary kidney	
		Acute impaired kidney function	
		Bilateral obstruction	
		Unmanageable symptoms	
		Normal kidney function	Non-deferrable (4–6 weeks)
		No solitary kidney	
		No infection	
	Ureteral or renal stone	Indwelling ureteral stent or nephrostomy tube	Semi-deferrable (6–12 weeks)
	Non-obstructing renal stone	Chronic impaired kidney function	Semi-deferrable (6–12 weeks)
		Solitary kidney	
		Normal kidney function	Deferrable (>12 weeks)
		No solitary kidney	
Functional urology	LUTS/BPH unresponsive to medical therapy	Indwelling transurethral catheter	Semi-deferrable (6–12 weeks)
		Indwelling suprapubic tube	
		Infection	
		Bladder stones	
		Diverticula	
		No indwelling transurethral catheter	Deferrable (>12 weeks)
		No indwelling suprapubic tube	
		No complications	
	Ureteral obstruction (non-stone-related)	Infection	Urgent (<24 h)
		Solitary kidney	
		Acute impaired kidney function	
		Bilateral obstruction	
		Unmanageable symptoms	
		Indwelling ureteral stent or nephrostomy tube	Semi-deferrable (6–12 weeks)
	Pelvic organ prolapse	Upper urinary tract obstruction	Semi-deferrable (6–12 weeks)
		Recurrent severe infection	
		No obstruction	Deferrable (>12 weeks)
		No infection	
	Urinary incontinence (male and female)		Deferrable (>12 weeks)
Andrology	Male infertility		Deferrable (>12 weeks)
	Testicular diseases (except for cancer)		Deferrable (>12 weeks)
	Penile diseases (except for cancer)		Deferrable (>12 weeks)
	Erectile dysfunction (surgical management)		Deferrable (>12 weeks)

LUTS/BPH = lower urinary tract symptoms suggestive of benign prostate hyperplasia.

The panel agrees with the option to offer immediate treatment in all cases of complicated obstructing ureteral or renal stones (infection, solitary kidney, bilateral obstruction, acute impaired kidney function).^7^ Moreover, the appropriate endourological treatment for non-complicated obstructing ureteral or renal stones should not be postponed, rather performed within 4–6 weeks.

Surgical treatment of patients with LUTS/BPH unresponsive to medical therapies should be considered semi-deferrable in case of complications (infection, bladder stones, diverticula) or indwelling transurethral catheter or suprapubic tube. Surgical treatment of patients with non-complicated LUTS/BPH can be considered deferrable according to the center waiting list.

The panel recommends that patients waiting for deferrable procedures should be periodically monitored with the appropriate exams in order to detect a possible clinical worsening. In that case, the procedure should be upgraded to semi-deferrable or non-deferrable.

### Outpatient procedures

The current panel position about outpatient procedures differs substantially from the previous one during the emergency phase.^1^ Strengthened by the efficacy of individual protection measures (use of masks and gloves, hand washing and social distancing)^8^ and the newly launched vaccination campaign, it is our view that all outpatient procedures can be regularly performed without substantial limitations, except for a mild reduction in the number of daily cases. Outpatient procedures include prostate biopsy, flexible cystoscopy, replacement of ureteral stent or nephrostomy tube as well as all diagnostic procedures for benign conditions (e.g. pressure-flow study). Also, intravesical therapies (BCG, Mitomycin C, others) for the treatment of high- or intermediate-risk non-muscle-invasive bladder cancer should be regularly performed.

## Proposed management of urological conditions in SARS-CoV-2-positive patients

Urologists practicing in hospitals treating COVID-19 patients may be in need to perform urgent procedures or consultation on infected patients. Although Ling et al.^9^ reported the presence of in the urine of 6.9% of the convalescent patients, in most other studies no single case of viral shedding in urine was documented.^10^ However, all health care workers should follow national and hospital rules in order to decrease the risk of contagion. Urgent surgical procedures on SARS-CoV-2 positive patients should be performed in dedicated operating rooms in adherence to clinical pathways implemented by the single hospitals. Considering the urgent procedures indicated in Table 1, the panel suggests that in SARS-CoV-2 positive patients with upper urinary tract obstruction, placement of a nephrostomy tube under local anesthesia is to be preferred to a ureteral stent using general anesthesia.

The increasing number of patients who are testing SARS-CoV-2 positive has opened a discussion about the optimal pathway for elective surgical treatment of urological malignancies or complicated benign conditions in infected, asymptomatic patients. The panel agrees that these patients should be appropriately scheduled and treated as soon as possible after test negativization.

## Surgical approach, surgical techniques, and new technologies

The panel agrees that in the current pandemic phase, the use of standardized surgical techniques in order to reduce the operating room time and the risk of postoperative complications is no longer recommended, but only suggested. Moreover, the panel recommends that all procedures should be performed by experienced surgeons or under their tutorship in the context of modular training programs. Implementation of new technologies as well as specific clinical studies on new technologies should be carefully considered, and applied only after local ethical committee approval.

At the beginning of the COVID-19 pandemic contrasting opinions have emerged about safety in the utilization of laparoscopy procedures (conventional and robot-assisted) as a consequence of the potential risk of dissemination of SARS-CoV-2 via laparoscopy gas. Whereas the Intercollegiate General Surgery Guidance recommended that laparoscopy should not be used,^11^ guidelines from EAU Robotic Urology Section provided a list of non-deferrable or semi-deferrable robot-assisted procedures to be performed based on the different impact of the COVID-19 pandemic across countries and hospitals.^12^ According to a systematic review of literature by the panel, it was concluded that specific clinical studies are needed to investigate the effective presence of the virus in the surgical smoke of different surgical procedures, and its concentration, and that, meanwhile, the adoption of all the required protective measures is mandatory.^13^ Moreover, we still suggest the implementation of the set of the intraoperative measures proposed by Zheng et al.^14^ (prevention of aerosol dispersal, lowering pneumoperitoneum pressure, lowering electrocautery power setting, use of bipolar cautery), as reported in the previous recommendation.^1^

## General organization and multidisciplinary management

In agreement with previous indications,^1^ the panel recommends the implementation of a multidisciplinary team of surgeons, anesthesiologists and operating room personnel who assign the most appropriate priority to patients, taking into account the availability of all resources necessary to activate the operating rooms. A pool of surgical procedures to be prioritized should be identified with proper planning on a weekly basis, and the possibility to accomplish them should be verified daily.

According to currently adopted protective measures, all patients scheduled for prioritized surgical procedures should be preoperatively tested for SARS-CoV-2 with a nasopharyngeal swab. The same should apply to patients requiring an urgent procedure, if the procedure can be deferred until the test result is available. The accuracy of rapid nasopharyngeal tests remains questionable. Nevertheless, it is always recommended that all patients had their temperature measured immediately before hospitalization, in order to prevent the hospitalization of suspected cases directly in the urological wards. At the time of hospitalization, all patients should wear a mask. Moreover, all urological departments in which logistics are suboptimal (i.e. one-bed rooms or larger two-bed rooms) should reduce the number of beds in order to increase the distance among patients.

The panel suggests considering the opportunity to screen patients for SARS-CoV-2 immediately before discharge, too.

Finally, as far as the complex management of patients with genitourinary malignancies is regarded, the implementation of virtual multidisciplinary meetings based on locally available web technologies is still recommended.

## Perioperative pathways for patients scheduled for elective surgery

In agreement with our previous recommendations,^1,15^ preoperative examinations in patients scheduled for elective surgery should be performed with a single hospital access whenever possible, after a negative telephone triage for COVID-19 symptoms, and using preferential and well-defined hospital pathways. Routine exams to define the anesthesiological risk and a nasopharyngeal swab for SARS-CoV-2 should always be performed before surgery, whereby it is advisable to guarantee a single access point to facilitate screening procedures in accordance with the indications of the Center for Disease Control and Prevention.^8^ After ruling symptoms and signs of COVID-19 out, hospitalized patients should wear a surgical mask and observe the hygiene rules recommended for the general population. The recommendation to use individual protection systems is mandatory both for patients and for healthcare workers.

Although patients following urologic surgical procedures should be discharged when their clinical conditions and logistics allow for a safe return home, the panel encourages an early discharge so as to reduce the potential risk of nosocomial infections (including COVID-19) and increase the bed availability for other patients. Anyway, patients discharged with indwelling drains or urinary catheters should be trained for completely autonomous home care. With regard to this, photo or video tutorials could be of aid for patients and their relatives to ease home care of urinary stoma. Patients discharged with indwelling internal ureteral stents need no specific advice. Moreover, using absorbable material for skin suture avoids subsequent hospital visits for their removal. We believe it is useful that ward staff provide as many instructions as possible during the hospital stay in order to facilitate home care. Telemedicine could be implemented to monitor those patients with a proper frequency during the early postoperative course according to their clinical needs.

Informing patients on the results of a pathology examination, especially after procedures for known or suspected genitourinary malignancies, has been one of the most critical points in the clinical pathway during the early phase of the pandemic. Contrary to the emergency phase, we now recommend that the pathology report should be discussed in the context of a scheduled face-to-face follow-up visit, in view of the current medico-legal restrictions and privacy-related issues.

## Future perspectives

There are two main areas of development for future scenarios. First, the reorganization of outpatient and inpatient urological activities determined by the current pandemic has the potential to favor the implementation of telemedicine. In a recent systematic review of the literature, Novara et al.^16^ reported a quite successful use of telehealth in selected settings of patients with prostate cancer, urinary incontinence, pelvic organ prolapse, non-complicated urinary stones, and urinary tract infections in non-pandemic times. It was, however, concluded that more studies are needed, especially to test the role of telehealth on other highly prevalent urological malignant and benign conditions. Considering that more robust data on long-term efficacy, safety, and health economics of telemedicine are mandatory, the panel suggests that at least the adoption of popular tools such as laptops, tablets, smartphones, or emails should be recommended for telemonitoring. Urology wards should implement systems for priority communications via telephone or email from patients to the medical or nurse staff in order to check the clinical course and decrease the risk of inappropriate hospital visits.

Second, we have just entered the era of vaccination against SARS-CoV-2, with the approval of vaccines that have been shown to be effective in pivotal phase III clinical trials by health authorities in many countries of the world.^5,6^ This will surely represent an immediate advantage in decreasing the risk of contagion among health workers with a positive impact on all medical activities, especially those performed in COVID-19 areas. Moreover, vaccination could mark the restart of didactic activities, such as training, courses, laboratory activities, and others. Finally, the possibility to track vaccinated patients could allow to progressively increase the currently held back urological activities, hence decreasing waiting lists. Although mass vaccination is a big step forward in the battle against COVID-19, it is still premature to predict over which time horizon, the anticipated effect of decreasing contagion and/or disease severity will be achieved. Therefore, the impact of vaccination on the reorganization of outpatient and inpatient urological activities might not be evident before 8–12 months from the start of the vaccination campaign. At the end of the vaccination phase, a further adaptation of the currently updated recommendations will be needed. Specifically, criteria to reschedule the surgical procedures that have been suspended in the past and current phases of the pandemic as well as resources to accomplish this challenging objective should be defined.

## Conclusions

The present white paper is the first update of a previous document published in the emergency phase of the COVID-19 pandemic, and is based on the opinions and experience of a group of urologists directly involved in the organization of some urological wards in Italy. An adaptation of the recommendations provided in the emergency phase is needed because numerous changes have characterized the urology practice in the last months, and several countries in the world are experiencing a second or even a third wave of contagion. The present update will hopefully be a valid tool to be used in the clinical practice and, possibly, a cornerstone for further discussion on the topic, also considering further evolution of the pandemic after the recently initiated mass vaccination campaign.
